# ALDH1A1 provides a source of meiosis-inducing retinoic acid in mouse fetal ovaries

**DOI:** 10.1038/ncomms10845

**Published:** 2016-02-19

**Authors:** Josephine Bowles, Chun-Wei Feng, Kim Miles, Jessica Ineson, Cassy Spiller, Peter Koopman

**Affiliations:** 1 Institute for Molecular Bioscience, The University of Queensland, Brisbane, Queensland 4072, Australia

## Abstract

Substantial evidence exists that during fetal ovarian development in mammals, retinoic acid (RA) induces germ cells to express the pre-meiotic marker *Stra8* and enter meiosis, and that these effects are prevented in the fetal testis by the RA-degrading P450 enzyme CYP26B1. Nonetheless, the role of RA has been disputed principally because germ cells in embryos lacking two major RA-synthesizing enzymes, ALDH1A2 and ALDH1A3, remain able to enter meiosis. Here we show that a third RA-synthesizing enzyme, ALDH1A1, is expressed in fetal ovaries, providing a likely source of RA in the absence of ALDH1A2 and ALDH1A3. In ovaries lacking ALDH1A1, the onset of germ cell meiosis is delayed. Our data resolve the conundrum posed by conflicting published data sets and reconfirm the model that meiosis is triggered by endogenous RA in the developing ovary.

## Abstract (web summary)

Recent findings have challenged the established concept that retinoic acid (RA) induces foetal germ cells to enter meiosis. Here, Bowles et al. identify the enzyme ALDH1A1 as a source of ovarian RA that may induce meiosis even when other RA-synthetic enzymes are deleted.

## Graphical abstract

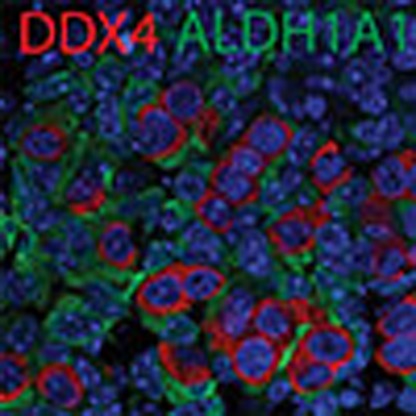

The question of how the sex-specific differentiation of germ cells is regulated during fetal development in mammals remains controversial. Until the mid-1970s, it was commonly believed that germ cell fate was genetically determined—that is, that XX germ cells were programmed to develop as oocytes and XY germ cells as sperm. Observation of germ cells in XX-XY chimeras disproved this concept by showing that XY germ cells could become oocytes^1^, implicating somatically derived secreted signals to either induce meiosis (in the ovary) or delay it until puberty (in the testis). Accordingly, experiments in which mouse fetal testes and ovaries were co-cultured each side of a permeable filter suggested that ovaries secreted a meiosis-inducing substance and testes secreted a meiosis-preventing substance^2^. However, observation of the fate of germ cells that failed to migrate correctly into the gonads suggested instead that all germ cells were programmed to initiate meiosis and enter the oogenic pathway unless prevented from doing so by a non-diffusible inhibitor of meiosis produced in the testis^3^.

The discovery that retinoic acid (RA) can regulate germ cell entry into meiosis appeared to resolve the controversy and provide a molecular basis for the control of germ cell differentiation^4^,^5^. RA is present at high levels in the mesonephros, adjacent to the developing gonads, presumably as a result of the strong expression there of ALDH1A2, the principal enzyme involved in the final step of RA synthesis^6^ and possibly ALDH1A3, a weaker RA-synthesizing enzyme^7^. From there it is thought to diffuse into the testis or the ovary. In the ovary, RA induces the pre-meiotic marker gene *Stra8* and markers of more advanced meiosis such as *Sycp3* and *Dmc1* (refs 4, 5, 8, 9). In the testis, the RA-degrading P450 enzyme CYP26B1 is highly expressed, clearing endogenous RA and preventing germ cells from being exposed to its influence^4^,^5^,^10^. Critical observations supporting these conclusions are that germ cells in fetal ovaries of vitamin A-deficient rat embryos fail to enter meiosis^11^, whereas in fetal testes of mouse embryos deficient in CYP26B1, germ cells enter meiosis ectopically^4^,^5^,^10^. The weight of evidence supporting regulation of entry into meiosis by a balance of retinoid synthesis and catabolism is reviewed elsewhere^12^.

Apparently conflicting with this model, a published report by Kumar *et al.* describes upregulation of *Stra8* and entry into meiosis by ovarian germ cells in mouse lines deleted for either *Aldh1a2* alone or in combination with the additional RA-synthesis gene *Aldh1a3* (ref. 13). The same authors induced meiosis in mouse fetal testes using the P450 inhibitor ketoconazole, but only when cultured together with the adjacent mesonephros. The authors contended that RA is therefore not involved in meiotic initiation, and suggested that an unidentified substance that originates in the mesonephros and is degraded by CYP26B1, but that is not synthesized by ALDH, triggers meiosis. They pointed to difficulties in visualizing RA in the developing ovary as further evidence for this opposing theory^13^,^14^.

We aimed here to address these apparent discrepancies and to further substantiate the role of RA as the principal driver of meiotic entry in mouse fetal ovaries. We show that RA is indeed present in the fetal ovary and that it becomes detectable in the fetal testis in the absence of CYP26B1. We show that ectopic *Stra8* expression and meiosis in the *Cyp26b1*-knockout testis is dependent on RA signalling. Critically, we demonstrate that a third RA-synthesizing enzyme, ALDH1A1, is expressed in the fetal ovary, that it produces RA, that expression of meiosis markers is delayed in its absence and that its expression is likely to be upregulated in the absence of ALDH1A2/3. Therefore, all available evidence suggests that onset of meiosis in the mouse fetal ovary is triggered by RA, with ALDH1A1 contributing to the sex-specific balance of RA levels in developing ovaries.

## Results

### RA is detectable in the developing ovary

Previous analyses of RA signalling activity in mouse fetal gonads using the transgenic RARE-lacZ reporter strain^15^ revealed strong staining in the mesonephroi and a lack of staining in the testes, as expected based on the expression of *Aldh1a2* and *Cyp26b1* (refs 4, 16). Surprisingly, in view of the apparent importance of RA in driving germ cell meiosis in the ovary, only very weak staining was observed in that tissue^4^,^5^. Nonetheless, evidence that only low levels of RA are present in the developing ovary is not inconsistent with the theory that RA induces *Stra8* expression in fetal ovarian germ cells because we and others have shown that isolated germ cells respond, in culture, to RA concentrations of <1 nM by upregulating *Stra8* (refs 8, 17).

**Figure 1:**
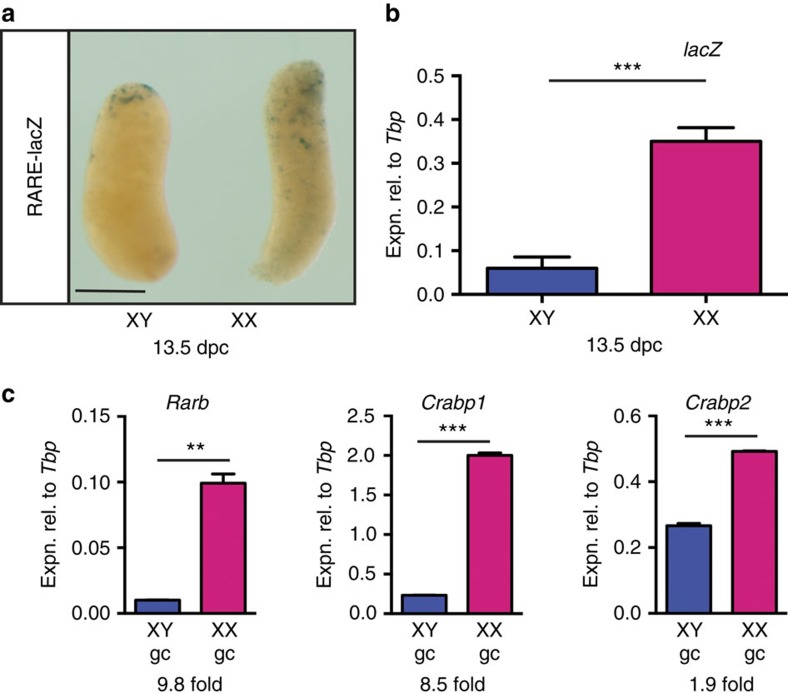
RA is detectable in the developing mouse ovary. (**a**,**b**) *LacZ* reporter gene activity (**a**) and expression (**b**) demonstrated in ovary (XX) but not in testis (XY) dissected from 13.5 d.p.c. RARE-lacZ transgenic embryos (for **b**, *n*=4, 6). (**c**) Expression of known RA target genes in germ cells isolated from 13.5 d.p.c. ovaries (XX) and testes (XY) at 13.5 d.p.c. (*n*=2). **P<0.01 and ***P<0.001 (Student's *t*-test, mean+s.e.m. is shown). Scale bar, 200 μm.

We reasoned that removing mesonephric tissue before assay might make the relative levels of staining in the testis and ovary easier to assess. Although staining remained weak in the developing ovary, it was clearly stronger than in the developing testis at the same time point (Fig. 1a and Supplementary Fig. 1). A small ‘cap' of strongly staining cells was commonly seen at the anterior poles of gonads of both sexes, and in some samples an anterior bias was observed in the overall staining intensity. Both phenomena may be related to the connection of the gonad to the mesonephric tubules at the anterior pole. It is unclear why RA was not detected in fetal ovaries in the Kumar *et al.*^13^ study. The RARE-lacZ transgene is prone to silencing, especially if kept in the homozygous state or if moved from the CD1 background (see Methods for details) so it is possible that the line used in that study was less sensitive than ours.

We next quantified the expression of *lacZ* in RARE-lacZ gonads by quantitative reverse transcription (qRT)–PCR, and found it to be significantly higher in the developing ovary compared with testis at 13.5 d.p.c. (days post coitum) (Fig. 1b, *P*=0.0002, unpaired *t*-test). Hence, this sensitive method of measuring lacZ expression confirms that endogenous RA is present in the developing ovary, albeit at relatively low levels.

Finally, we tested whether known RA-responsive genes were more highly expressed in ovarian germ cells, relative to testicular germ cells, as a readout of RA exposure. To that end, we isolated germ cells from 13.5 d.p.c. ovaries and testes by fluorescence-activated cell sorting (FACS) and measured the expression of *RARb*, *Crabp1* and *Crabp2.* Expression was higher in ovarian germ cells by 9.8-, 8.5- and 1.9-fold, respectively (Fig. 1c), suggesting that RA is present at levels sufficient to drive expression of target genes.

### Stra8 expression in the Cyp26b1-knockout testis depends on RA

**Figure 2:**
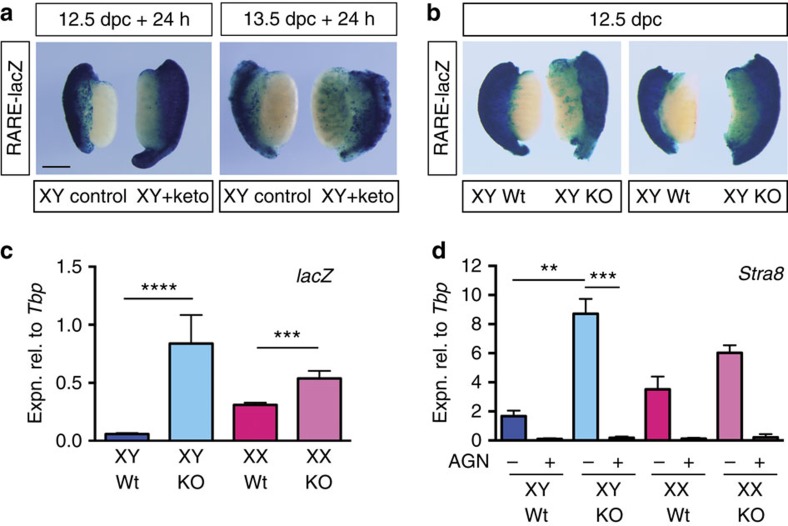
CYP26B1 degrades endogenous RA in the developing testis. (**a**) Urogenital ridges (UGRs, gonad plus mesonephros) dissected at 12.5 or 13.5 d.p.c. from RARE-lacZ transgenic embryos were cultured for 24 h in the presence of ketoconazole (0.7 μM) and stained to visualize RA signalling. (**b**,**c**) 12.5 d.p.c. UGRs from RARE-lacZ;*Cyp26b1* wild-type (Wt) or RARE-lacZ;*Cyp26b1* knockout (KO) embryos were stained to visualize RA signalling (**b**) or lacZ expression was measured by qRT–PCR of gonad-only tissue (**c**, *n*=22, 6, 20, 3). (**d**) To demonstrate that ectopic induction of *Stra8* in the *Cyp26b1*-knockout testis requires RA signalling, 11.5 d.p.c. UGRs were dissected from *Cyp26b1* wild-type (Wt) or knockout (KO) embryos and cultured for 50 h in the presence or absence of AGN193109 (AGN) and *Stra8* expression was measured by qRT–PCR (*n*=6, 6, 7, 7, 3, 3, 2, 2). **P<0.01, ***P<0.001, ****P<0.0001 (Student's *t*-test, unpaired for **c**, paired for **d**, mean+s.e.m. shown). Scale bar, 200 μm.

RA was previously shown to be increased threefold in *Cyp26b1*-knockout testes relative to wild type, using the indirect assay of incubating tissue extracts with a reporter cell line^10^. We confirmed this result using the direct assay system of RARE-LacZ reporter mice. When 12.5 or 13.5 d.p.c. RARE-lacZ gonads with attached mesonephroi were cultured with the P450 inhibitor ketoconazole for 24 h and then stained for β-galactosidase activity, more intense staining was observed in treated XY samples than in untreated controls (Fig. 2a). This result contrasts with that reported in Kumar *et al.*^13^ in which no change to detectable RA was found after 48 h culture in ketoconazole. It is unclear why our findings differ from theirs: it is possible that our RARE-lacZ line is more sensitive than theirs, that endogenous RA is no longer detectable after 48 h in culture or that their experiment was compromised in some other way.

To validate these results genetically, we crossed the RARE-lacZ transgenic and *Cyp26b1*-knockout^18^ mouse lines and stained for β-galactosidase activity in 12.5 d.p.c. testes. Staining was substantially increased in the absence of *Cyp26b1* (Fig. 2b). qRT–PCR showed that *lacZ* expression was ∼12-fold higher in the *Cyp26b1*-knockout testes than in wild-type testes (Fig. 2c). Together, these results confirm that CYP26B1 degrades endogenous RA in the fetal testis.

We next addressed the issue of whether ectopic meiosis in *Cyp26b1*-knockout testes^4^,^10^ is driven by ectopic (undegraded) RA or whether an undegraded non-RA CYP26B1 substrate might be responsible, as has been suggested^13^. We cultured 11.5 d.p.c. *Cyp26b1*-knockout testes for 50 h in the presence or absence of the potent pan retinoic acid receptor (RAR) antagonist AGN193109 (ref. 19). Treatment with this compound did not induce general toxicity in these cultures (Supplementary Fig. 2). As expected, *Stra8* expression was substantially elevated in *Cyp26b1*-knockout testes compared with wild-type testes, in the absence of antagonist (Fig. 2d). However, RAR antagonist completely eliminated *Stra8* upregulation, indicating that the expression of *Stra8* in the absence of CYP26B1 is dependent on RAR signalling in this system, thus implicating RA and opposing the theory of a non-RA meiosis inducer^13^.

### Aldh1a1 is expressed and produces RA in the developing ovary

**Figure 3:**
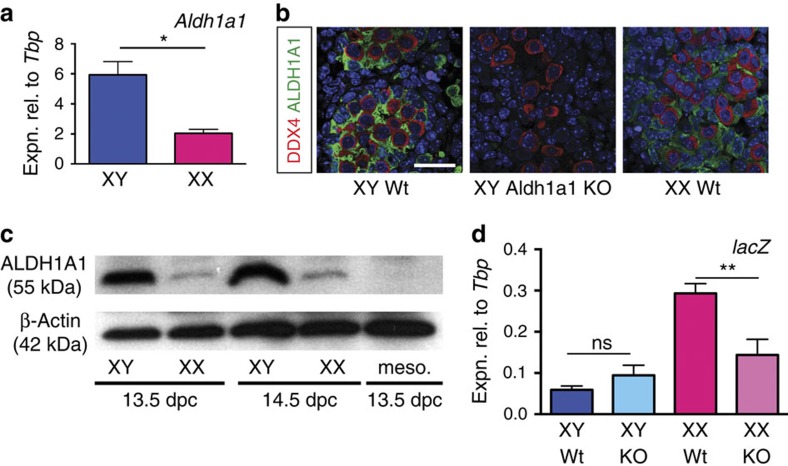
*Aldh1a1* is expressed and ALDH1A1 catalyses RA production in the fetal mouse ovary. (**a**) *Aldh1a1* expression was measured by qRT–PCR in gonad-only tissue dissected at 13.5 d.p.c. from C57BL/6 embryos (*n*=3,3). (**b**) By immunofluorescence ALDH1A1 expression is lower in fetal ovary (XX WT) than in fetal testis (XY Wt) but is detectable above background levels (XY ALDH1A1 KO) at 14.5 d.p.c. DDX4 (red) marks germ cells. Scale bar, 25 μm. (**c**) By western blot analysis, ALDH1A1 is detectable in fetal ovaries (XX) at lower levels than in fetal testes (XY) at 13.5 and 14.5 d.p.c. but is not detected in fetal mesonephros (meso.). (**d**) Gonads from RARE-lacZ;ALDH1A1 wild-type (Wt) or RARE-lacZ;ALDH1A1-knockout (KO) embryos were isolated and lacZ expression was measured by qRT–PCR (*n*=18, 4, 14, 8). *P<0.05, **P<0.01 (Student's unpaired *t*-test, mean+s.e.m. shown).

We previously documented a high level of *Aldh1a1* expression during testicular development in the mouse^20^. In that study, we found comparatively low levels of expression in the ovaries of outbred Swiss albino (Quackenbush) mouse embryos. The reported entry of germ cells into meiosis in *Aldh1a2/a3* double knockout ovaries^13^ prompted us to consider whether ALDH1A1 might provide an alternative source of ovarian RA, and therefore to examine gonadal *Aldh1a1* expression in more detail and in a different mouse strain. We first re-examined *Aldh1a1* expression, this time on an inbred C57BL/6 background (because this is likely more similar to the background present in the *Aldh1a2/3* knockout studies than the outbred strain used in our initial studies), and found it to be ∼3-fold higher in the testis than the ovary at 13.5 d.p.c. (Fig. 3a). Others have also reported low but detectable expression of *Aldh1a1* in 13.5 d.p.c. ovary tissue, using section *in situ* hybridization^21^. Analysis of *Aldh1a1* expression in somatic and germ cell populations at 12.5 d.p.c. indicated that the gene is predominantly expressed by somatic cells, rather than germ cells (Supplementary Fig. 3). Further, ALDH1A1 protein was detectable by immunofluorescence in the developing ovary at 14.5 d.p.c. at levels higher than the background observed in XY *Aldh1a1*-knockout gonads^22^ processed in parallel (Fig. 3b). Finally, we used western blotting to test for the presence of ALDH1A1 protein in gonad samples taken at 13.5 and 14.5 d.p.c. We found that ALDH1A1 was present in ovarian samples at low but detectable levels; in contrast, we were unable to detect ALDH1A1 protein in mesonephros tissue (Fig. 3c) or in *Aldh1a1*-knockout retinal tissue collected at 14.5 d.p.c. (Supplementary Fig. 4). In sum, these results suggest that *Aldh1a1a1* mRNA and protein are present in developing ovaries, albeit at lower levels than in testes.

In view of our hypothesis, it was important to test the contribution of ALDH1A1 expression to the production of RA during ovarian development. To this end, we crossed the RARE-lacZ transgene into the *Aldh1a1*-knockout line and used qRT–PCR to assay *lacZ* mRNA levels at 13.5 d.p.c. Expression was significantly lower in the *Aldh1a1*-knockout than in wild-type ovaries (Fig. 3d, *P*=0.0021, unpaired Student's *t*-test). Thus, ovarian ALDH1A1 contributes substantially to the RA content of the fetal ovaries, likely in addition to mesonephric ALDH1A2 and -3.

### Entry into meiosis is delayed in Aldh1a1-knockout ovaries

**Figure 4:**
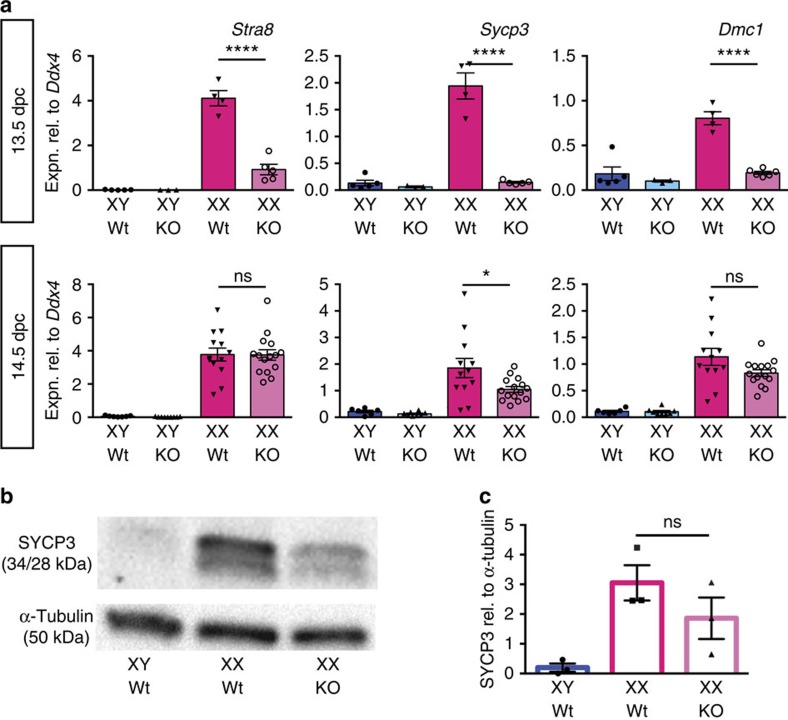
Meiotic onset is delayed in the absence of ALDH1A1. (**a**) Gonads were collected from *Aldh1a1*-knockout (KO) or wild-type littermates (Wt) at 13.5 or 14.5 d.p.c. and subjected to qRT–PCR analysis for expression of the pre-meiotic gene *Stra8* or meiotic genes *Sycp3* and *Dmc1* (*n*=5, 3, 4, 5 for 13.5 d.p.c. and 6, 8, 12, 15 for 14.5 d.p.c.). Meiotic onset was delayed at 13.5 d.p.c. but had largely recovered by 14.5 d.p.c. (**b**,**c**) Western blot analysis demonstrated that less SYCP3 protein was detectable in 14.5 d.p.c. gonad tissue of XX *Aldh1a1*-knockout (KO) compared with XX wild-type (Wt) littermates (*n*=3, 3, 3). NS, not statistically significant, *P<0.05, ****P<0.0001 (Student's unpaired *t*-test, mean±s.e.m. shown).

We next determined whether germ cell entry into meiosis was affected in *Aldh1a1*-knockout ovaries. Complete blockade of meiosis was not expected because *Aldh1a1*-knockout adult females are fertile^22^, and because ALDH1A2 and -3 would still be active and producing RA in the mesonephros. Using qRT–PCR, we measured expression of *Stra8*, *Sycp3* and *Dmc1* in 13.5 and 14.5 d.p.c gonads of wild-type and *Aldh1a1*-knockout embryos (Fig. 4a). At 13.5 d.p.c., expression of all markers was significantly lower in the *Aldh1a1* XX samples (*P*<0.0001; Student's unpaired *t*-test). By 14.5 d.p.c., *Stra8* and *Dmc1* expression was normal, and *Sycp3* expression had largely recovered. Western blotting of 14.5 d.p.c. gonadal extracts showed that lower levels of SYCP3 protein were present in *Aldh1a1*-knockout ovaries relative to wild type (Fig. 4b,c). The temporal profile of marker expression suggests a delay rather than an impairment of meiosis and, combined with the above data, these results indicate that ALDH1A1 produces some of the RA in the ovary that triggers meiotic onset *in vivo*.

### Aldh1a1 expression is sensitive to RA levels in the testis

**Figure 5:**
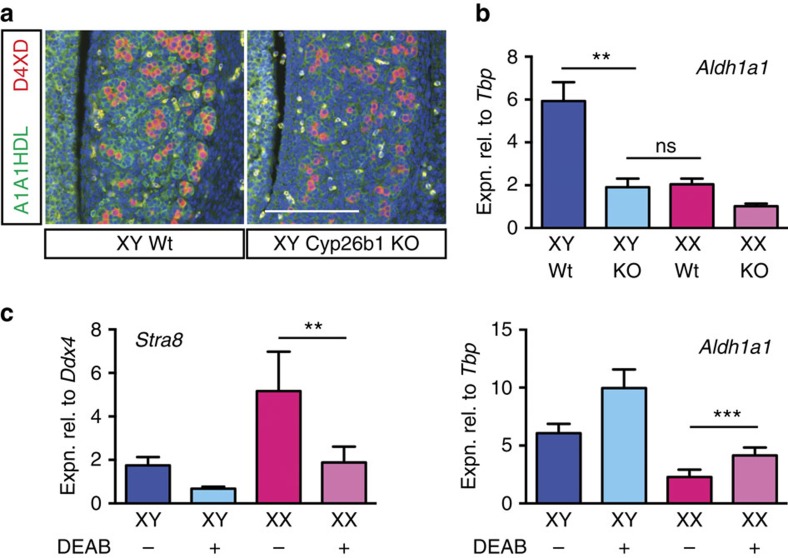
ALDH1A1 expression responds to levels of RA present. (**a**,**b**) Immunofluorescence (**a**) and qRT–PCR (**b**) analyses show ALDH1A1/*Aldh1a1* expression in 13.5 d.p.c. fetal testis is diminished when *Cyp26b1* is deleted (for b, *n*=3, 5, 3, 2), scale bar, 100 μm. (**c**) When 11.5 d.p.c. UGRs are cultured in the presence of DEAB (ALDH inhibitor) for 48 h *Stra8* expression is diminished, as expected, but *Aldh1a1* expression is augmented in ovary suggesting that in *Aldh1a2/3*-knockout ovaries *Aldh1a1* expression may be elevated above normal levels (*n*=2, 2, 5, 7). NS, not statistically significant, **P<0.01, ***P<0.001 (Student's *t*-test, unpaired for **b**, paired for **c**, mean+s.e.m. shown).

It is well established that expression of RA synthesis and degradation enzymes is sensitive, to some extent, to the presence of RA^23^ and we previously postulated that high *Aldh1a1* expression in the developing testis is a response to, and a read-out of, the efficient clearing of endogenous RA by CYP26B1 (ref. 20). To test whether ALDH1A1 expression responds to RA availability in our system, *in vivo*, we examined ALDH1A1 mRNA and protein production in gonads of 13.5 d.p.c. *Cyp26b1*-knockout embryos, which have elevated levels of endogenous RA (Fig. 2). Both mRNA and protein were substantially downregulated when *Cyp26b1* was deleted (Fig. 5a,b), with *Aldh1a1* levels in XY *Cyp26b1*-knockout testes similar to those in the XX wild-type ovaries (Fig. 5b). We also tested this effect in urogenital ridge (UGR) culture and found that there was a dose-dependent diminution of *Aldh1a1* expression in the presence of exogenously added RA and that this is mirrored by an increase in expression of *Cyp26b1* (Supplementary Fig. 5).

Given that an increase in RA availability is associated with a loss of ALDH1A1 expression in our system, we wondered if the converse was also true. Specifically, we reasoned that loss of ALDH1A2/3, with associated loss of RA production, might lead to increased *Aldh1a1* expression. Such a scenario could help explain the fact that, at least to some extent, *Stra8* is still expressed and germ cells still enter meiosis in the ALDH1A2/3 null model^13^. As we do not have access to *Aldh1a2* and *Aldh1a3*-knockout mouse lines, we tested the effect of blocking ALDH-mediated RA production by treatment of gonadal cultures with the ALDH inhibitor N,N-diethylaminobenzaldehyde (DEAB)^24^,^25^. Previous studies have demonstrated that DEAB treatment adversely affects expression of *Stra8* and meiosis markers as well as entry into meiosis^26^. After culturing 11.5 d.p.c. UGRs (C57BL/6) for 2 days in the presence of DEAB, we found that *Aldh1a1* was indeed upregulated in treated gonadal tissue, whereas the control gene, *Stra8*, was downregulated, as expected (Fig. 5c). Together, these results show that *Aldh1a1* expression is sensitive to RA levels in the developing gonads, and suggest the possibility that the amount of ALDH1A1 available to supply RA to stimulate germ cell meiosis in the developing ovary might be enhanced in the *Aldh1a2/3* double knockout.

## Discussion

We designed and conducted this study to address the question of whether or not RA is required to initiate meiosis in the mouse fetal germ cells, given that conflicting data exist on this issue. A great deal of evidence supports the view that RA directly triggers *Stra8* expression and the onset of meiosis (reviewed in ref. 12). The strong upregulation of meiotic markers in *Cyp26b1*-knockout fetal testes provides genetic evidence that RA is sufficient to drive germ cells to enter meiosis^4^,^10^. In addition, the finding that ovarian germ cells in vitamin A-deficient rat embryos do not enter meiosis but, instead, remain undifferentiated^11^ indicates that RA is necessary and not just sufficient. In contrast, one study was unable to detect ovarian RA, and found that at least some germ cells express *Stra8* and enter meiosis despite the absence of two RA-synthesizing enzymes, ALDH1A2 and ALDH1A3; it was suggested that the meiosis inducer is a CYP26B1 sensitive, but non-RA substance^13^. The present study settles this apparent conundrum by directly addressing several unresolved issues.

First, we confirmed that endogenous RA is present in the developing ovary, albeit at relatively low levels. Because *all-trans* RA can trigger *Stra8* expression in purified germ cell cultures at concentrations as low as 1 nM (refs 8, 17), the observed low levels of RA detected in the ovary are consistent with RA triggering *Stra8* expression. Our findings contrast with those of Kumar *et al.*^13^, who were unable to find any evidence of endogenous RA in the developing ovary. It is known that the reporter mouse line RARE-lacZ is prone to lose sensitivity with time and/or when bred onto some backgrounds; it may be that an RARE-lacZ mouse stock of sub-optimal sensitivity was used in some previous studies.

Second, we demonstrated that the ectopic *Stra8* expression observed in the *Cyp26b1*-knockout testis results from ectopic RA signalling rather than some other non-RA mechanism as has been suggested^13^. Although this point was important to establish experimentally, it is perhaps not surprising given that, to our knowledge, no non-retinoid substrate for CYP26B1 has been identified.

Finally, we showed that ovarian ALDH1A1 has a role in this system. Our data show that ALDH1A1 is present in the developing ovary and that, in its absence, detectable RA is diminished in that tissue at 13.5 d.p.c. Accordingly, meiosis, as judged by the expression of key marker genes, was delayed in the *Aldh1a1*-knockout ovary, but still occurred, consistent with the observation that *Aldh1a1*-knockout female mice are fertile^22^. It seems highly likely, however, that RA from the mesonephros is also involved in inducing meiosis: first, the expression of meiotic markers in the *Aldh1a1*-knockout ovary is delayed not abolished and, second, an anterior to posterior wave of meiosis is observed^27^,^28^,^29^, consistent with the mesonephric duct and tubules being a source of at least some of the meiosis-inducing RA^16^.

It is clear that the activity of ALDH1A1 in this system provides a ready explanation for the published observation that ovarian germ cells remain able to enter meiosis in *Aldh1a2/3* knockout mice^13^—observations commonly regarded as undermining the role of RA in inducing meiosis in the fetal ovary^13^,^14^,^30^. Our observation that *Aldh1a1* expression is sensitive to RA suggests that *Aldh1a1* expression may be higher than normal in *Aldh1a2/3* knockout mice, a possibility we were not able to test in this study.

Other authors have addressed the question of whether the mesonephros is the only source of meiosis-inducing RA and identified ALDH1A1 as an enzyme potentially involved in the production of RA in the ovary tissue^26^. Our results are complementary to theirs but our conclusions differ in three respects. First, we do not consider that the ovary is the sole or major source of RA involved in meiotic induction, given that expression of meiotic genes was delayed but quickly recovered in the absence of *Aldh1a1*. We cannot support the assertion, in the previous study, that *Aldh1a1* is more highly expressed in the ovary than is *Aldh1a2* in the mesonephros, because simple qRT–PCR analyses do not allow comparison of levels of expression of different genes^26^. Finally, we do not find that ALDH1A1 is localized in the germ cells. In addition to the evidence we produce here, regarding this point, microarray expression data from sorted cell populations of fetal mouse gonads found that germ cells are the only cell type that does not express *Aldh1a1* in the fetal ovary^31^.

In summary, our demonstration that ALDH1A1 synthesizes detectable levels of RA in the developing ovary effectively quashes published concerns regarding the role of RA as the ovarian meiosis trigger^13^,^14^,^30^. The postulated non-RA meiosis inducer has not come to light, and we consider that there is little evidence for its existence. Our data align well with findings in the human fetal ovary where it is postulated that RA driving the initiation of meiosis is produced locally, rather than in the mesonephric tissue, and that the principal enzyme involved in this is ALDH1A1 (refs 32, 33).

## Methods

### Mice

All animal work was conducted according to protocols approved by the University of Queensland Animal Ethics Committee. RARE-lacZ transgenic mice, which harbour three copies of the RARβ2 RARE linked to the hsp68 minimal promoter^15^, were obtained from the Jackson Laboratory (Tg(RARE-Hspa1b/lacZ)12Jrt/J) and maintained heterozygous on a CD1 background as is recommended to prevent silencing of the transgene. Oct4ΔPE:eGFP (OG2) strains^34^, in which fetal germ cells are marked by eGFP expression, were a gift of Jeff Mann (mixed C57BL/6 and CD1 background). The *Aldh1a1*-knockout line^22^ (gift of Gregg Duester, available from the Jackson Laboratory, B6.129-Aldh1a1tm1Gdu/J) and the *Cyp26b1*-knockout line^18^ (gift of Hiroshi Hamada, available from the Jackson Laboratory, Cyp26b1tm1Hh) were both on a pure C57/BL6 inbred background. *Aldh1a1*-knockout genotyping used forward primer 5′-GATGTTTGGTCTTTACATTTTGGGC-3′ together with reverse primer 5′-GCACCAACACATTCTCTAACGTGA-3′ (producing a wild-type band of ∼800 bp) and KO forward primer 5′-ATCAGAAGCTTAAGGATCCACTCG-3′ together with reverse primer as before, producing a deletion band of ∼250 bp. Wild-type embryo tissues used in cultures with DEAB were C56BL/6 (ARC, Western Australia). Noon on the day on which the mating plug was observed was designated 0.5 d.p.c. Embryo sex was determined by PCR of tail tissue^35^.

### Whole mount lacZ staining

Fresh gonadal or cultured UGR (gonad plus mesonephros) tissue was stained for β-galactosidase activity using standard procedures and incubating overnight at 37 °C (ref. 36).

### Germ cell isolation

Testis and ovary tissues were collected from Oct4ΔPE:eGFP matings at 12.5 or 13.5 d.p.c. and dissociated and germ cell (GFP+) and somatic cell (GFP−) fractions were isolated by FACS using a FACSAria Cell Sorter (BD Biosciences).

### Quantitative RT–PCR

Freshly dissected or cultured gonad or UGR samples, or freshly isolated cells, were subjected to RNA synthesis (Qiagen RNeasy micro kit including DNase treatment) and cDNA synthesis (Applied Biosystems (ABI), High Capacity cDNA Archive kit). Relative cDNA levels were determined by the 2^−ΔCT^ method with reactions including Taqman PCR master mix (ABI) and Taqman gene expression sets and carried out on ABI Prism 7000, 7500 or ViiA7 machines. Control genes used for normalization were *Tbp* (encoding the ubiquitously-expressed TATA box binding protein) or *Ddx4* (*Mvh*, encoding a germ cell-specific marker). Taqman gene expression sets were: *Tbp* (Mm00446973_m1), *Ddx4* (Mm00802445_m1), *lacZ* (Mr03987581_mr), *Aldh1a1* (Mm00657317_m1), *Stra8* (Mm00441558_m1), *Sycp3* (Mm00488519_m1), *Dmc1h* (Mm00494485_m1), *Rarb2 (Mm01319677_m1)*), *Crabp1* (Mm00442776_m1), *Crabp2* (Mm00801693_g1) and *Cyp26b1* (Mm00558507_m1).

### Statistics

Statistical significance was determined using Prism GraphPad software. Data are presented as mean+s.e.m. When UGR or gonad pairs were split to provide control and culture samples, Student's paired (two-tailed) *t*-test was used (Figs 2d and 5c). If samples were not paired, Student's unpaired (two-tailed) *t*-test was used for two-way comparisons. Asterisks highlight the pertinent comparisons and indicate level of statistical significance (*P<0.05; **P<0.01; ***P<0.001; ****P<0.0001; NS=not statistically significant).

### UGR culture

For ketoconazole (0.7 μM, Sigma Aldrich, ethanol vehicle), AGN193109 (5 μM, Vitae Pharmaceuticals, DMSO vehicle) or DEAB (50 μM, Sigma Aldrich, DMSO vehicle) culture, UGRs (gonad with mesonephros attached) were dissected at the appropriate time point from RARE-lacZ, *Cyp26b1*-knockout;RARE-lacZ, *Cyp26b1*-knockout or wild-type (C56BL/6) embryos, the pairs were separated into control (vehicle only) and treated and individually cultured in 40 μl hanging drops in media (DMEM (Invitrogen) with 10% FBS (AusGenex), penicillin (Invitrogen, 50 U ml^−1^) and streptomycin (Invitrogen, 50 μg ml^−1^)). To prepare hanging drops, UGRs were washed through a pool of control (with vehicle) or supplemented media, removed in a 40 μl volume and deposited in the centre of circles on the lid of a 24-well culture plate. Each well was filled with 500 μl of PBS before the lid was inverted. All cultures were held in a 37 °C incubator at 5% CO_2_.

### Section immunofluorescence

Whole embryos were recovered at the appropriate time point and section immunofluorescence (IF) analyses were carried out on fixed, paraffin-embedded 7 μm sections using standard methods as detailed previously^17^ with high pH antigen retrieval and blocking in 10% heat-inactivated horse serum in PBTX (phosphate-buffered saline with 0.1% Triton X-100 detergent). Primary antibodies used were: mouse anti-DDX4 (1/400, anti-DDX4/MVH, ab27591, Abcam), rabbit anti-ALDH1A1 (1/200. ab24343, Abcam). Secondary antibodies (from Molecular Probes, Invitrogen) were goat anti-mouse IgG Alexa Fluor 594 and goat anti-rabbit IgG Alexa Fluor 488 all used at 1:200 dilution. Slides were imaged on an Olympus BX-51 fluorescence microscope.

### Western blot

Western blots were performed by standard methods on whole gonad-only tissue with two gonads used per lane or, as a control, on mesonephros-only tissue or eye tissue. Antibodies used were rabbit anti-ALDH1A1 (1/200, ab24343, Abcam), mouse anti-β-actin (Thermo Scientific, PIEMA5-15739, 1:5000), rabbit anti-SYCP3 (1:200, anti-SCP3, ab15093, Abcam), mouse anti-α-tubulin (Sigma Aldrich, T5168, 1:5,000), goat anti-mouse conjugated to horseradish peroxidase (Sigma; 1:2,000) and goat anti-rabbit conjugated to horseradish peroxidase (Vector Laboratories; 1:2,000). Briefly, gonad pairs were dissociated with a 13-G needle and lysed in 1 × SDS sample buffer (62.5 mM Tris-HCl (pH 6.8), 2% SDS, 10% glycerol, 50 mM dithiothreitol and 0.01% (w/v) bromo-phenol blue), separated on SDS–PAGE and transferred to a polyvinylidene difluoride membrane (Millipore). Membranes were incubated for 2 h at room temperature with primary antibody and then overnight at 4 °C. After membrane rinsing, immunoreactive proteins were visualized using Clarity Western ECL Substrate (Bio-Rad) on a ChemiDoc machine (Bio-Rad). For relative quantification of SYCP3 protein, raw intensity of bands was determined using Image Lab Software (version 4.0). SYCP3 intensity units were calculated relative to α-tubulin loading control and relative expression was calculated for three independent sets of gonad samples (XY Wt control, XX Wt control and XX knockout taken from the one litter) on each of three independent blots. Error bars represent s.e.m. calculated from the three independent biological replicates.

## Additional information

**How to cite this article:** Bowles, J. *et al.* ALDH1A1 provides a source of meiosis-inducing retinoic acid in mouse fetal ovaries. *Nat. Commun.* 7:10845 doi: 10.1038/ncomms10845 (2016).

## Supplementary Material

Supplementary Information Supplementary Figures 1-5
