# Supplementary material for: ALDH1A1 provides a source of meiosis-inducing retinoic acid in mouse fetal ovaries
Source: Nat Commun. 2016 Feb 19;7:10845. doi: 10.1038/ncomms10845 (PMC4762892; doi:10.1038/ncomms10845)
Supplement: Supplementary Information — Supplementary Figures 1-5 [file ncomms10845-s1.pdf]

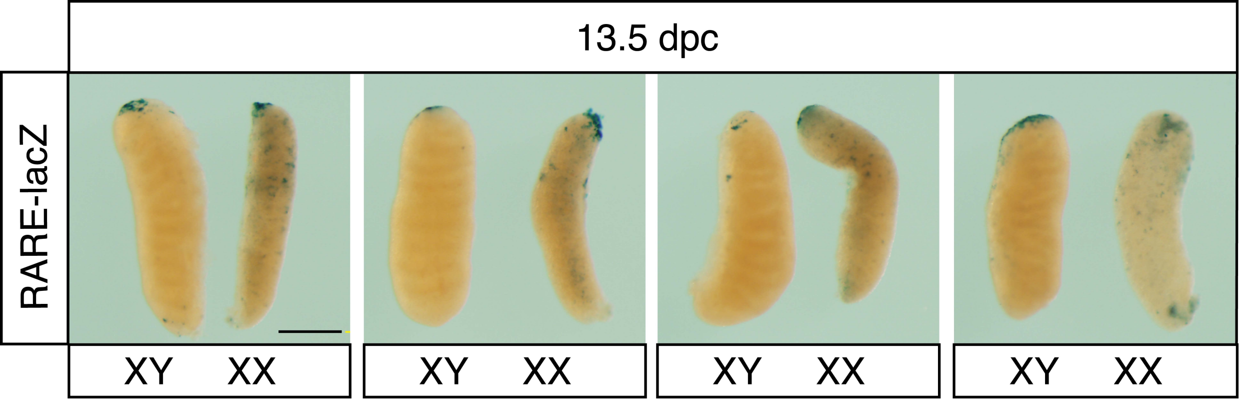

Supplementary Figure 1.
Retinoic acid (RA) is detectable in the developing mouse ovary. LacZ reporter gene activity demonstrated in ovary (XX) but not testis (XY) dissected from 13.5 dpc RARE-lacZ transgenic embryos. RARE; RA response element. Scale bar, 20 μm.

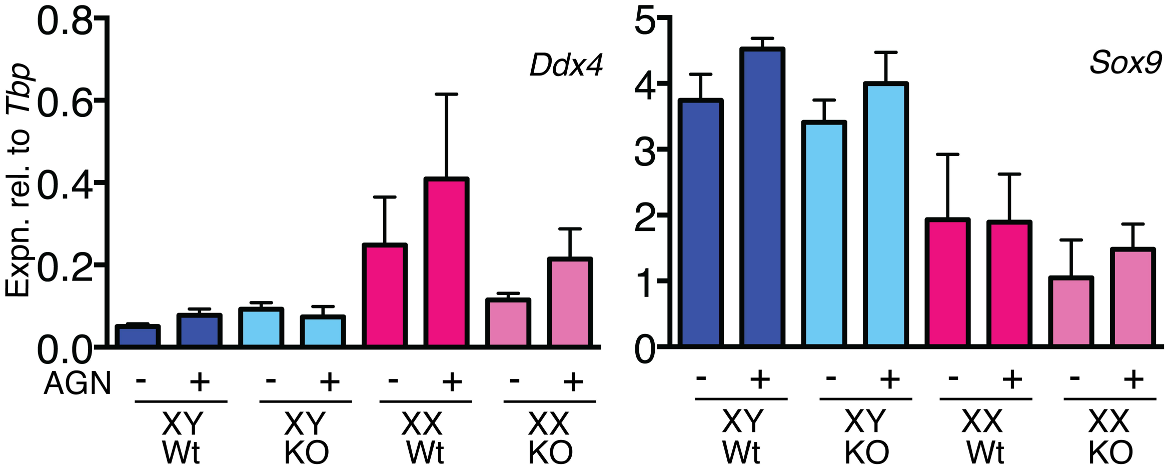

Supplementary Fig. 2.
Culture in RA receptor antagonist does not induce generalized toxicity in cultures.
11.5 dpc urogential ridges were dissect from wild type (Wt) or *Cyp26b1* knockout (KO) embryos and cultured for 50 hours in the presence or absence of a pan RA receptor antagonist, AGN193109 (AGN) and *Mvh* and *Sox9* expression were measured by qRT-PCR (mean + S.E.M.; n=6,6,7,7,3,3,2,2).

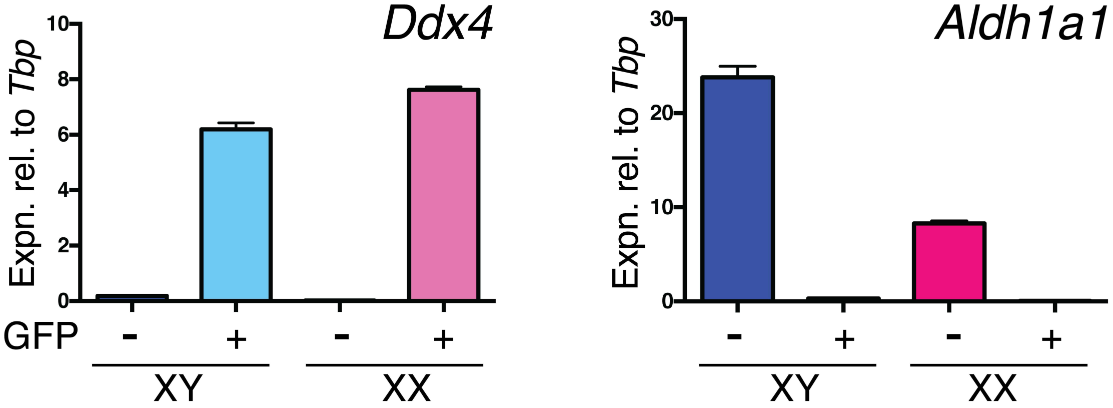

Supplementary Figure 3.

***Aldh1a1* is predodminantly expressed by somatic cell and not germ cells.** Somatic cells (GFP-) and germ cells (GFP+) were isolated by fluorescence-activated cell sorting (FACS) from 12.5 dpc testes (XY) and ovaries (XX) of the Oct4ΔPE:eGFP line and subjected to quantitative reverse transcription PCR (qRT-PCR). Near absence of Ddx4 (*Mvh)* expression in somatic pools demonstrates that there is very little germ cell contamination. Most *Aldh1a1* expression is observed in the somatic pool demonstrating that germ cells do not express appreciable levels of *Aldh1a1*. Mean + S.E.M. is shown for technical replicates (n = 3). GFP; green fluorescent protein.

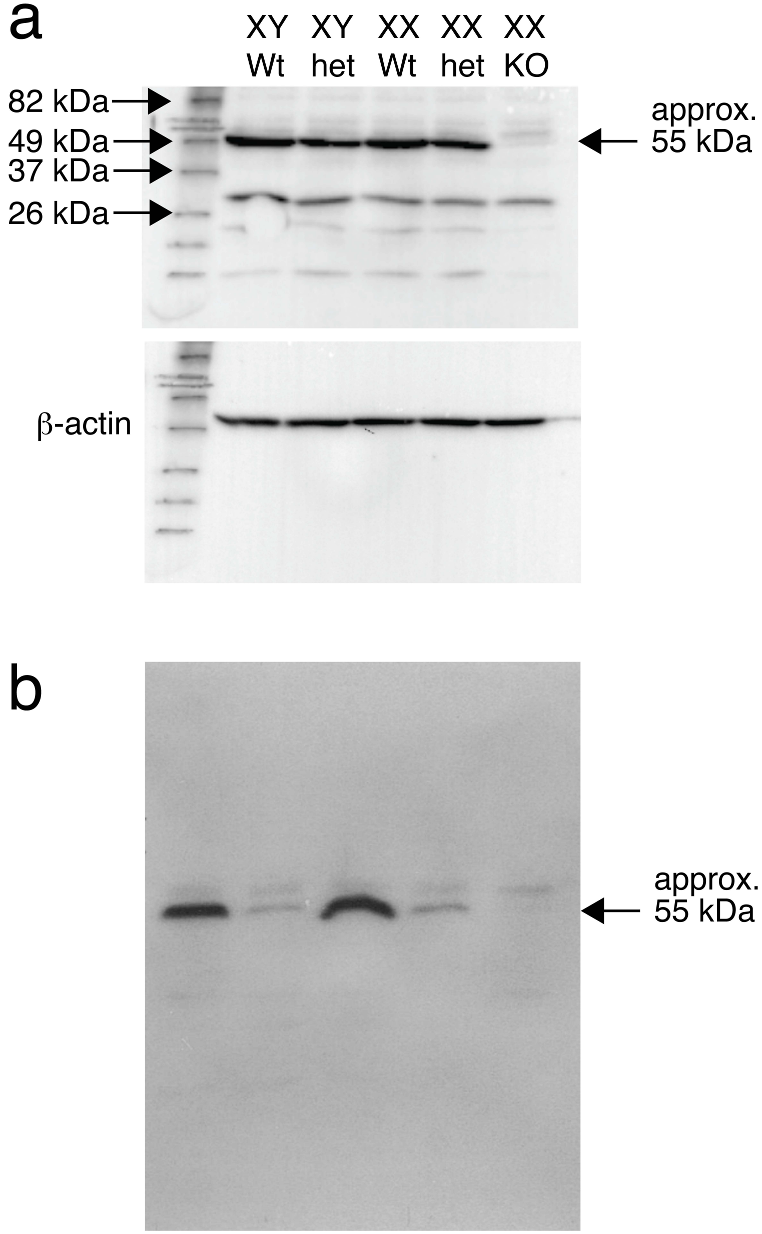

Supplementary Fig. 4.
(a) Anti-ALDH1A1 antibody is specific for a band of approximately 55 kDa. By Western blot analysis a band of approximately 55 kilodaltons (kDa) is detected in wildtype eye tissue but not in retinal tissue of XX KO embryo at 14.5 dpc. The blot was reprobed with anti-β-actin antibody to demonstrate similar loading of all lanes. The predicted molecular weight for Aldh1a1 is 55 kDa. The marker used is BenchMark Pre-Stained Protein Ladder (LifeTechnologies) (b) Full blot of the ALDH1A1 panel shown in Fig. 3c. In gonadal tissue extracts the anti-ALDH1A1 antibody detects one major band that is not detected in mesonephric tissue.

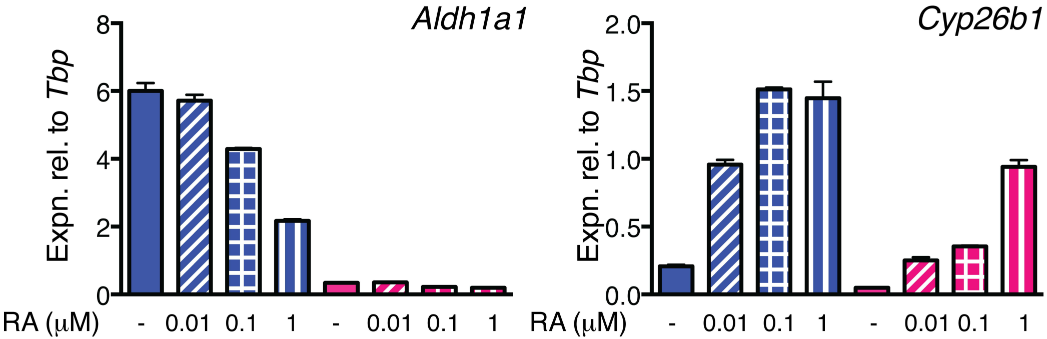

Supplementary Figure 5.

***Aldh1a1* expression responds to levels of RA present.** 11.5 dpc urogential ridges (UGRs) were cultured for 22 hours in the presence of varying doses of all-trans RA as indicated. Each culture included 6 or 7 UGRs from distinct embryos. Increasing concentration of RA induced *Cyp26b1* expression and diminished *Aldh1a1* expression. Error bars (mean + S.E.M.) report technical replicates (n = 3).
